# Novel Electrochemiluminescence Sensor for Dopamine Detection Based on Perylene Diimide/CuO Nanomaterials

**DOI:** 10.3390/molecules30010184

**Published:** 2025-01-05

**Authors:** Qirong Tian, Xinyang Sun, Chuan Li, Lei Shang, Rongna Ma, Xiaojian Li, Liping Jia, Shuijian He, Qian Zhang, Wei Zhang, Huaisheng Wang

**Affiliations:** 1 School of Chemistry and Chemical Engineering, Liaocheng University, Liaocheng 252059, China; 2310120234@stu.lcu.edu.cn (Q.T.); y2861698401@163.com (X.S.); lichuan@lcu.edu.cn (C.L.); shanglei@lcu.edu.cn (L.S.); marongna@lcu.edu.cn (R.M.); lixiaojian@lcu.edu.cn (X.L.); or jialiping@lcu.edu.cn (L.J.); hswang@lcu.edu.cn (H.W.); 2 Co-Innovation Center of Efficient Processing and Utilization of Forest Resources, International Innovation Center for Forest Chemicals and Materials, College of Materials Science and Engineering, Nanjing Forestry University, Nanjing 210037, China; shuijianhe@njfu.edu.cn; 3 Co-Innovation Center of Efficient Processing and Utilization of Forest Resources, International Innovation Center for Forest Chemicals and Materials, College of Science, Nanjing Forestry University, Nanjing 210037, China

**Keywords:** perylene diimide derivative, CuO, dopamine sensor, electrochemiluminescence resonance energy transfer, self-assembly

## Abstract

Dopamine (DA) is an important catecholamine neurotransmitter and its abnormal concentration is closely related to diseases such as hypertension, Parkinson’s disease and schizophrenia. Due to the advantages of high sensitivity and fast response for electrochemiluminescence (ECL), developing ECL sensors for detecting DA was very critical in clinical diagnosis. ECL resonance energy transfer (ECL-RET) was an effective signaling mechanism. However, the shortage of highly efficient ECL-RET pairs impeded the development of DA sensors. Herein, methyl-modified perylene diimide derivative (PDI-CH_3_) self-assembly nanorod materials as luminophores and CuO nanomaterials as acceptors were integrated into nanocomposites. An obvious ECL-RET was found in PDI-CH_3_/CuO nanocomposites. After PDI-CH_3_/CuO nanocomposites were treated with DA, a large increase in ECL intensity was observed. Then, PDI-CH_3_/CuO nanocomposites were taken as an ECL platform to detect DA. This ECL sensor exhibited a linear response to DA from 10^−12^ M to 10^−8^ M with a limit of detection of 0.20 pM. Compared with other sensors for DA detection, the constructed ECL sensor exhibited higher sensitivity. In addition, the novel ECL sensor in this work showed good practicability in a human serum sample.

## 1. Introduction

Dopamine (DA) [1] is an important catecholamine neurotransmitter and its abnormal concentration is closely related to diseases such as hypertension, Parkinson’s disease and schizophrenia [2,3,4]. Thus, being able to detect its concentration sensitively was critical in clinical diagnosis [5]. Many technologies such as colorimetry (CL) [6,7], fluorescence (FL) [8,9] and electrochemistry (EC) [10,11] have been used for designing sensors [12,13] to detect DA. Compared with the optics technology (CL and FL) that requires an expensive spectrometer and high background, EC technology has the advantages of low cost, fast response, low background, high sensitivity and attracted more interest [14]. Electrochemiluminescence (ECL) [15] is a technique that combines chemiluminescence (CL) and EC. The luminescence is produced by the luminophores triggered at EC potential, which do not require an expensive spectrometer like CL and FL, having the advantages of EC such as low cost, fast response and possessing lower background and higher sensitivity than that of EC. Therefore, developing sensitive ECL sensors for DA detection was important [16].

Recently, many reported ECL sensors were depending on resonance energy transfer (RET) [17,18] to quench ECL signals, which has been taken as an effective signaling mechanism. A good ECL-RET system requires overlap between the absorption spectrum of the acceptor and the emission spectrum of the donor (luminophores) and close proximity (<10 nm) between the acceptor and the donor. Most ECL-RET sensors separate the donor and acceptor by the aptamer [19] or the antigen/antibody [20,21]. The long energy transfer path would result in great energy loss and low ECL efficiency. To enhance the ECL-RET efficiency, integrating the acceptor and donor into a nanocomposite [22] was proposed to develop an ultrasensitive ECL sensor. It opened a new window to obtain good ECL-RET systems and construct ultrasensitive sensors. Therefore, screening new matched ECL-RET donor–receptor pairs was urgent.

More efforts were devoted to developing novel ECL luminophores with low triggering potential and long wavelength light [23,24,25,26]. This was because the low triggering potential could efficiently eliminate the background interference from excited oxygen at a highly negative potential [27,28,29] (<−1.0 V) and largely improve the sensitivity of sensors. In addition, the long wavelength light and low triggering potential could avoid the destruction of biomolecules and greatly enhance the accuracy of sensors. Recently, some perylene diimide derivatives (PDIs) have been drawn more attention due to their low triggering potential [30,31,32,33] (about −0.5 V) with ECL wavelengths around 700 nm [34,35]. And various metals and metal oxides such as AuNPs [36], Ag [37], ZnO [38] and MnO_2_ [39] were often combined with ECL luminophores to design sensors. CuO nanomaterials [40,41] possessed wide absorptions between 250 nm and 700 nm, which overlapped with the ECL spectra of PDIs and were good acceptors.

**Scheme 1:**
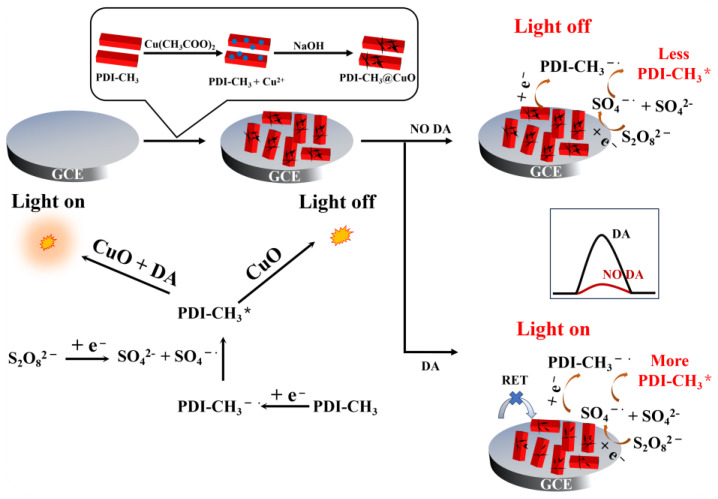
The ECL detection mechanism for DA based on PDI-CH_3_/CuO nanosmaterials.

Herein, we integrated methyl-modified PDIs (PDI-CH_3_) together with CuO nanomaterials as an ECL platform for DA detection. PDI-CH_3_ self-assembly material emitted strong ECL at −0.33 V with coreactant K_2_S_2_O_8_. The ECL (wavelength between 580 nm and 700 nm) could be quenched by CuO nanomaterials through the ECL-RET mechanism. In addition, CuO nanomaterials covered on the PDI-CH_3_ self-assembly material also impeded the effective collision between PDI-CH_3_ and K_2_S_2_O_8_ and decreased the ECL intensity. After PDI-CH_3_/CuO_-_modified GCE was reacted with DA, CuO nanomaterials were reduced into Cu^+^ due to the reductive property of DA [22]. Then, CuO nanomaterials were reduced, ECL-RET decreased and the effective collision between PDI-CH_3_ and K_2_S_2_O_8_ increased, which led to the recovery of ECL. The difference in ECL intensity was taken as a detection signal for DA (Scheme 1).

## 2. Results and Discussion

### 2.1. The Property Characterization of PDI-CH_3_/CuO Nanomaterials

**Figure 1:**
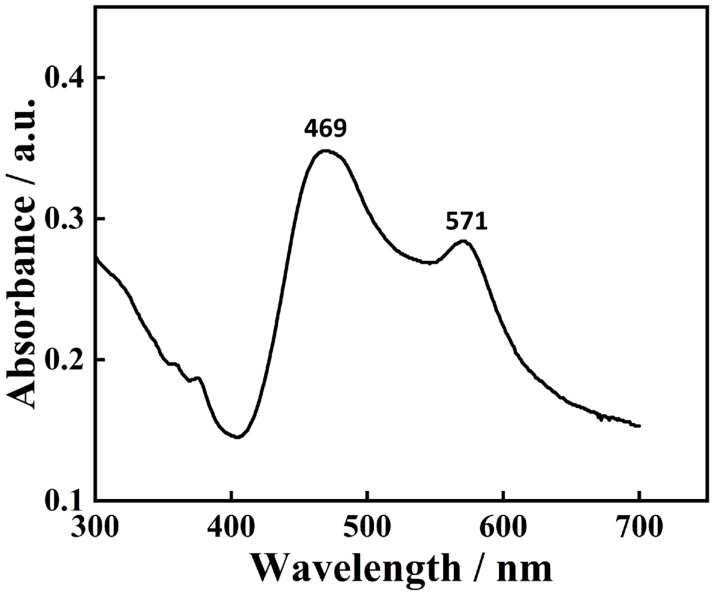
The UV–Vis spectra of PDI-CH_3_ solution.

The PDI-CH_3_ molecules consisted of a perylene unit and N-methyl dicarboximides. The UV–Vis spectrum of the PDI-CH_3_ aqueous solution showed a wide absorption band of the perylene unit at 468 nm and 571 nm [42,43] (Figure 1). The absorption band at 468 nm was integrated by two absorption bands of A^0–2^ and A^0–1^ and the absorption band at 571 nm was from A^0–0^. The integrated absorption bands and the low ratio of A^0–0^/A^0–1^ indicated that PDI-CH_3_ molecules aggregated.

**Figure 2:**
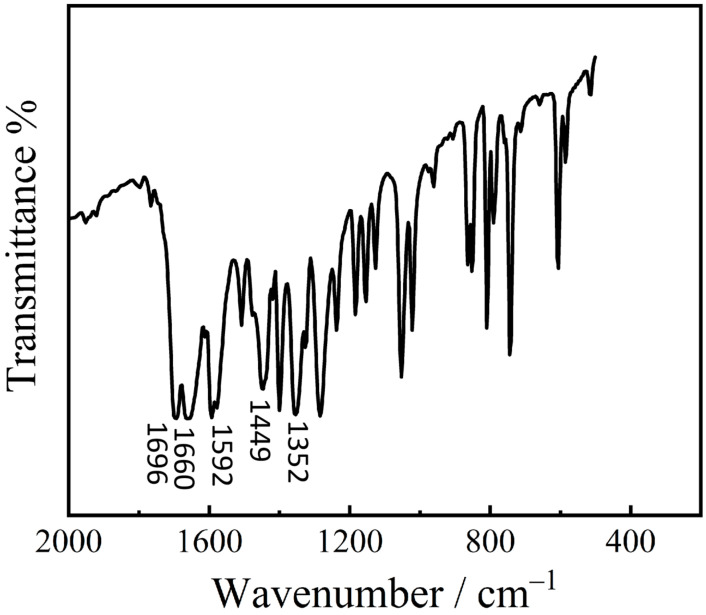
The IR spectra of PDI-CH_3_.

**Figure 3:**
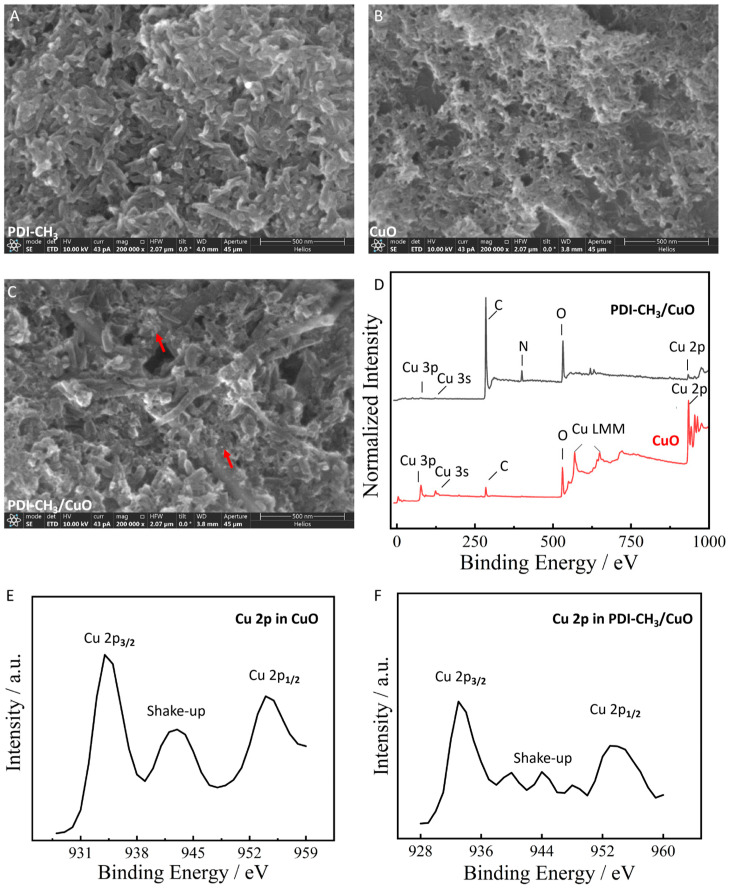
The SEM image of PDI-CH_3_ (**A**), CuO (**B**) and PDI-CH_3_/CuO (**C**). (**D**) The XPS of CuO (red line) and PDI-CH_3_/CuO (black line). The XPS spectra of Cu 2p in CuO (**E**) and the Cu 2p in PDI-CH_3_/CuO (**F**).

The infrared (IR) spectrum (Figure 2) of the PDI-CH_3_ molecules showed the vibration band of C=O at 1696 cm^−1^, N-C=O at 1660 cm^−1^, C=C at 1592 cm^−1^ and CH_3_ at 1449 and 1352 cm^−1^. The short nanorod structure of the PDI-CH_3_ molecules self-assembled through π–π stack interaction, and hydrogen bonds were observed on the SEM images (Figure 3A). The prepared CuO nanomaterials were needle-like nanostructures, as shown in Figure 3B. In the PDI-CH_3_/CuO hybrid nanomaterial, a needle-like nanostructure was found on the PDI-CH_3_ nanorods, indicating the successful preparation of the PDI-CH_3_/CuO hybrid nanomaterial (Figure 3C). The XPS spectra of CuO and PDI-CH_3_ were also recorded (Figure 3D). The Auger spectrum of Cu (metallic copper, LMM) was reported at about 567 eV, and the Auger spectra of oxidized Cu(II) and Cu(I) (LMM) were reported at about 650 eV and 570.6 eV, respectively [44]. The XPS spectra of the CuO nanomaterial showed the peaks of Cu 3p at 77.2 eV, Cu 3s at 123.5 eV, C 1s at 286.0 eV, O 1s at 531.6 eV Cu LMM-1 Auger peak at 569.5 eV and Cu LMM-2 Auger peak at 649 eV and Cu 2p at 934.6 eV (Figure 3D, red plot), which indicated that copper existed in the form of oxides. The high-resolution spectra of Cu 2p (Figure 3E) showed the Cu 2p_3/2_ peak at 934.0 eV and Cu 2p_1/2_ at 954.0 eV. The 20 eV energy gap was consistent with the standard spectra of CuO [45]. In addition, there was an obvious shake-up satellite peak (942.9 eV) between 940 eV and 950 eV, indicating the presence of CuO [46]. The XPS spectra of PDI-CH_3_/CuO hybrid nanomaterial showed the obvious peaks of Cu 3p at 77.2 eV, Cu 3s at 123.5 eV, C 1s at 286.0 eV, N 1s at 400.0 eV, O 1s at 532.8 eV and Cu 2p at 934.7 eV (Figure 3D, black plot), in which the peaks of Cu 3p, Cu 3s and Cu 2p were attributed to the CuO nanomaterial. The N 1s peak at 400.0 eV was attributed to PDI-CH_3_. In addition, the high-resolution spectra of Cu 2p in PDI-CH_3_/CuO (Figure 3F) showed the Cu 2p_3/2_ at 933.0 eV, Cu 2p_1/2_ at 953.0 eV and the shake-up satellite peak at 944 eV, demonstrating the presence of CuO. The above results demonstrated the successful preparation of the PDI-CH_3_/CuO hybrid nanomaterial.

### 2.2. The ECL Detection Principle of DA Sensors

**Figure 4:**
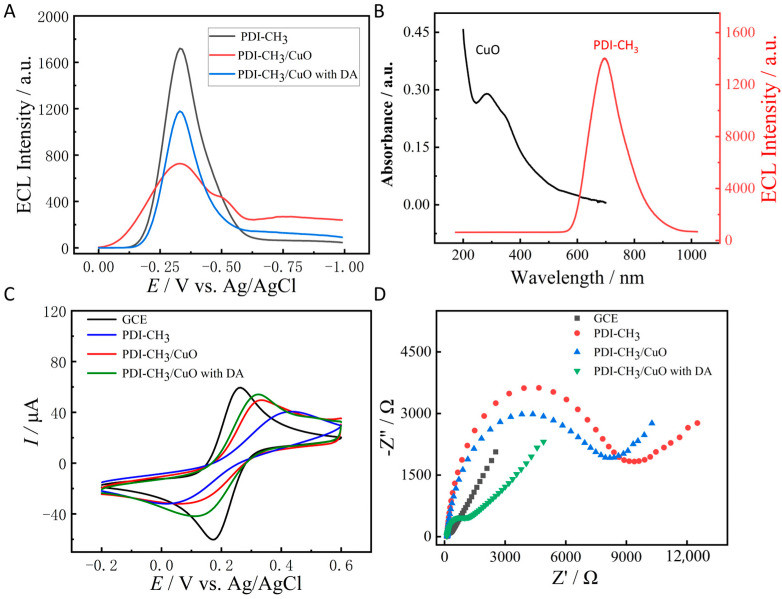
(**A**) The ECL intensity of PDI-CH_3_-modified GCE (black line), PDI-CH_3_/CuO-modified GCE (red line) and PDI-CH_3_/CuO-modified GCE treated with DA (blue line) recorded in 0.1 M PBS (pH 7.0) with 0.1 M K_2_S_2_O_8_ during the potential scanning range from 0 to −1.0 V. (**B**) The UV–Vis spectra of CuO solution (black line) and the ECL spectra of PDI-CH_3_-modified GCE recorded in 0.1 M PBS (pH 7.4) with 0.1 M K_2_S_2_O_8_ during the potential scanning range from 0 to −1.0 V (red line). The CV (**C**) and EIS (**D**) of bare GCE (black line), PDI-CH_3_-modified GCE (red line), PDI-CH_3_/CuO-modified GCE (blue line) and PDI-CH_3_/CuO-modified GCE treated with DA (green line) performed in 0.1 M Na_2_SO_4_ (pH 6.0) with 5 mM K_3_Fe[CN]_6_/K_4_Fe[CN]_6_.

The ECL curves of PDI-CH_3_ and PDI-CH_3_/CuO hybrid nanomaterials-modified GCE were, respectively, recorded in 0.1 M PBS solution with 0.15 M K_2_S_2_O_8_ when the cyclic voltammetry (CV) potential was scanned from 0 to −1.0 V. The PDI-CH_3_-modified GCE produced strong ECL at −0.33 V (Figure 4A, black plot), which was attributed to the coreaction of PDI-CH_3_ and S_2_O_8_^2−^. S_2_O_8_^2−^ was electrochemically reduced to SO_4_^−^ and SO_4_^2−^ when the CV potential was scanned from 0 to −1 V^23^. PDI-CH_3_ was also electrochemically reduced to PDI-CH_3_^−^ when the CV potential was scanned from 0 to −1 V. Then, the reduced PDI-CH_3_ (PDI-CH_3_^−^) reacted with SO_4_^−^ (reduced S_2_O_8_^2−^) and produced excited PDI-CH_3_ (PDI-CH_3_*), emitting ECL with a wavelength at 696 nm according to the ECL spectra recorded in Figure 4B, as shown by the red line. After CuO nanomaterials were grafted on PDI-CH_3_ self-assembly nanorods, the ECL intensity significantly decreased (Figure 4A, red plot). This was because there was part of an overlap between the absorption band of CuO (Figure 4B, black line) and the ECL emission spectra of PDI-CH_3_ (Figure 4B, red line). The distance between CuO and PDI-CH_3_ was so short that the efficient ECL-RET in the PDI-CH_3_/CuO nanocomposite took place. In addition, CuO impeded the approach of S_2_O_8_^2−^ to PDI-CH_3_, produced less PDI-CH_3_* and decreased the ECL intensity. When PDI-CH_3_/CuO-modified GCE was immersed in the DA solution, the ECL intensity increased (Figure 4A, blue plot). This was because two hydroxyl groups attached to a benzene ring of DA could be recognized by CuO nanomaterials. Then, CuO was reduced to Cu^+^ by DA, and DA was oxidized to dopamine-o-quinone (DOQ), leading to the fall off of CuO from GCE. This decreased the ECL-RET effect, allowed more S_2_O_8_^2−^ to react with PDI-CH_3_ and enhanced the ECL intensity.

The cyclic voltammetry (CV) (Figure 4C) and the electrochemistry impedance spectra (EIS) (Figure 4D) of different modification electrodes were also recorded in 0.1 M Na_2_SO_4_ (pH 6.0), including 5 mM K_3_Fe[CN]_6_/K_4_Fe[CN]_6_. There were reversible redox peaks for Fe[CN]_6_^3−^/Fe[CN]_6_^4−^ on bare GCE (Figure 4C, black line) and a small electron transfer resistance (*R*_et_) for bare GCE (Figure 4D, black square). The PDI-CH_3_-modified GCE showed a lower redox peak current and a larger difference in peak potential on the CV plot (Figure 4C, red line) and a larger *R*_et_ due to the bad conductivity of PDI-CH_3_ self-assembly nanorods [47,48] (Figure 4D, red dot). After CuO nanomaterials were grafted on PDI-CH_3_ self-assembly nanorods (Figure 4D, blue triangle), the redox peak current became larger and the difference in peak potential became smaller on the CV plot (Figure 4C, blue line). The *R*_et_ became smaller due to the enhanced conductivity of CuO on the EIS plot (Figure 4D, blue dot). After DA reacted with PDI-CH_3_/CuO nanomaterials, the redox peak current became larger and the difference in the peak potential became smaller on the CV plot (Figure 4C, green line). Also, the *R*_et_ largely decreased (Figure 4D, green inverted triangle). This was because CuO nanomaterials were reduced into Cu^+^. In addition, DA molecules adsorbed on the surface of PDI-CH_3_/CuO nanomaterials were protonated at pH 6.0. The Cu^+^ and protonated DA molecules created positive charges on the electrode surface, which attracted more negative Fe[CN]_6_^3−^/Fe[CN]_6_^4−^ to the electrode surface and accelerated the electron transfer [49].

### 2.3. The Optimization of ECL Sensors

**Figure 5:**
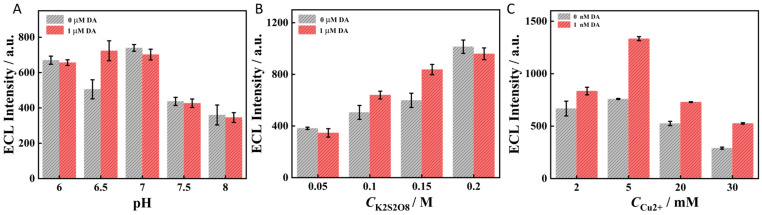
The optimized conditions: (**A**) pH of electrolyte solution with *C*_cu2+_ at 2 mM and *C*_K_2_S_2_O_8__ at 0.1 M; (**B**) *C*_K_2_S_2_O_8__ with *C*_cu2+_ at 2 mM and pH at 6.5; (**C**) *C*_cu2+_ with pH at 6.5 and *C*_K_2_S_2_O_8__ at 0.15 M.

To construct an optimized biosensor, the pH of the electrolyte solution and the concentration of Cu^2+^ and K_2_S_2_O_8_(*C*_Cu^2+^_ and *C*_K_2_S_2_O_8__) for preparing biosensors were optimized (Figure 5). When the *C*_Cu^2+^_ was at 2 mM and *C*_K_2_S_2_O_8__ was at 0.1 M, the pH from 6.0 to 8.0 was investigated (Figure 5A). The difference in the ECL intensity of the biosensor between 0 µM DA and 1 µM DA was the largest for pH at 6.5. Thus, 6.5 was taken as the optimized pH. When the pH was at 6.5 and *C*_Cu^2+^_ was at 2 mM, *C*_K_2_S_2_O_8__ from 0.05 to 0.2 M was tested (Figure 5B). The difference in the ECL intensity of the biosensor between 0 µM DA and 1 µM DA was the largest for *C*_K_2_S_2_O_8__ at 0.15 M. When pH was at 6.5 and *C*_K_2_S_2_O_8__ was at 0.15 M, the difference in the ECL intensity of the biosensor between 0 nM DA and 1 nM DA was the largest for *C*_Cu^2+^_ at 5 mM (Figure 5C).

### 2.4. The Property of ECL Sensor for DA Detection

**Figure 6:**
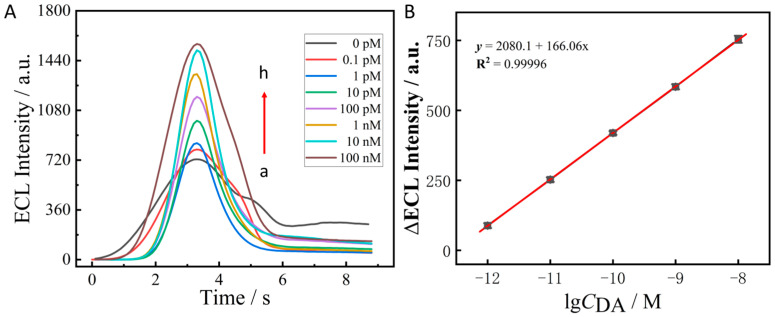
(**A**) The ECL intensity of biosensors at different *C*_DA_: 0 (a), 10^−13^ (b), 10^−12^ (c), 10^−11^ (d), 10^−10^ (e), 10^−9^ (f), 10^−8^ (g) and 10^−7^ (h) M. (**B**) The relationship between the difference in ECL intensity and lg*C*_DA_. The number of measurement repetitions at each concentration was three and the RSD% was from 1.5% to 9.9%.

The ECL sensors for DA detection were prepared at optimized parameters. With increasing *C*_DA_ from blank (Figure 6A, plot a) to 10^−7^ M (Figure 6A, plot h), the ECL intensity of biosensors increased drop-wisely. The ECL biosensors exhibited a good linear relationship between the ECL intensity and *C*_DA_ from 10^−12^ to 10^−8^ M (Figure 6B) with the equation ΔECL = 2080.1 + 166.06x and R^2^ 0.99996. The low limit of detection was 0.20 pM, which was calculated according to 3σ. Compared with other DA sensors [22,50,51,52,53] (Table 1), the constructed ECL sensors exhibited higher sensitivity.

### 2.5. The Reproducibility, Stability and Selectivity of ECL Sensor

**Figure 7:**
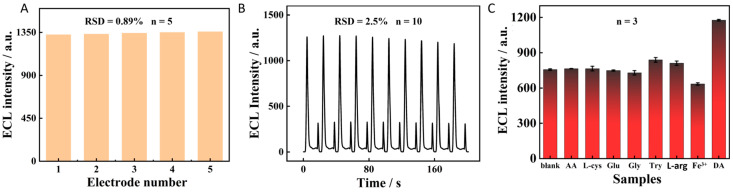
(**A**) The ECL intensity of five parallel DA sensors with *C*_DA_ 1 nM. (**B**) The ECL intensity of DA sensor for ten continuous cycles. (**C**) The ECL intensity of DA sensor for blank, AA, L-cys, Glu, Gly, Try, L-arg, Fe^3+^ (10 nM) and 0.1 nM DA. The number of measurement repetitions for every interference was three and the RSD% was from 0.27% to 2.6%.

To evaluate the properties of the sensors, reproducibility, stability and selectivity were important factors for consideration. Five DA ECL sensors were parallelly prepared (Figure 7A). The ECL intensity of these parallel sensors at 1 nM DA was similar and the RSD was 0.89%, demonstrating good reproducibility. Ten continuous cycles of ECL measurement of sensors at 0.1 nM DA were performed to test the stability of ECL biosensors. Their ECL intensity was similar and the RSD was 2.5%, indicating good stability of the ECL sensors (Figure 7B). The interferences such as ascorbic acid (AA), L-cysteine (L-cys), glucose (Glu), glycine (Gly), Tryptophan (Try), L-arginine (L-arg) and Fe^3+^ (all interferences at 10 nM) were used to test the selectivity of the ECL sensors. The Fe^3+^ could weaken the ECL signal of this system, which might be due to the chelation of Fe^3+^ and PDI-CH_3_/CuO. However, the ECL intensity of the sensors for these interferences was similar to that at blank, indicating good selectivity of the sensors (Figure 7C).

### 2.6. ECL Detection of DA in Human Serum Sample

The human serum sample from the volunteer was taken as a real sample to assess the feasibility of the constructed ECL sensor for DA detection. The DA solution with different concentrations was, respectively, added to the human serum sample. Then, the human serum samples were diluted 100 times for ECL detection by the constructed ECL sensor (see Table 2). The recovery rate was from 92.6% to 105.8% and the RSDs were less than 5%. The results demonstrated the good feasibility of the constructed ECL DA sensor in a human serum sample.

## 3. Materials and Methods

All ECL experiments were performed on a Model RFL-2 ECL analyzer (Xi’an Remex Instrument Co., Ltd., Xi’an, China) with electrochemical workstation CHI 660B (Shanghai CHI Instruments Co., Shanghai, China). The ECL spectra were recorded on the ECL spectroscopy were recorded on a Model ECL S-MK Spectrograph (FORTEC Technology (HK) Co., Ltd., Hongkong, China). The electrochemical impedance spectroscopy (EIS) was performed in a CHI 760C electrochemical workstation. The working electrode was glassy carbon electrode (GCE) (3 mm diameter), reference electrode was Ag/AgCl (KCl-saturated) and counter electrode was platinum wire.

The human serum sample was obtained from volunteer in the Dongchangfu People’s Hospital (Liaocheng, China). The volunteer has given informed consent. This study was performed in strict accordance with the International Ethical Guidelines for Biomedical Research Involving Human Subjects (WHO/CIOMS, 2002) and was approved by the Liaocheng University Institutional Review Board (IRB).

## 4. Experimental Section

### 4.1. Chemicals

N,N′-Dimethyl-3,4,9,10-perylenedicarboximide (PDI-CH_3_) (98%) was purchased from Sigma–Aldrich. L-cysteine (L-cys), glucose (Glu), glycine (Gly), Tryptophan (Try) and L-arginine (L-arg) were obtained from Shanghai Linc-Bio Science Co. Ltd. (Shanghai, China). Ascorbic acid (AA), FeCl_3_·6H_2_O, copper acetate (Cu(CH_3_COO)_2_), glacial acetic, sodium hydroxide (NaOH) and potassium persulfate were purchased from Aladdin Industry Corporation (Shanghai, China). The human serum sample was obtained from Dongchangfu People’s Hospital. Ultrapure water (>18 MΩ) from a Milli-Q Plus system (Millipore, Burlington, MA, USA) was used to prepare aqueous solutions.

### 4.2. Instruments

The scanning electron microscopy (SEM) images were recorded on Thermo Fisher Scientific FIB-SEM GX4 (Waltham, MA, USA). X-ray photoelectron spectroscopy (XPS) was measured on a Thermo Escalab Xi+ Spectrometer with a monochromated Al Kα X-ray resource, and C 1s at 284.4 eV was used as a reference for all binding energies. Ultraviolet–visible (UV–vis) absorption spectra were recorded on Lambda 750 spectrophotometer of Perkin Elmer in Shelton, CT, USA. The FT-IR spectrum was recorded on NICOLET 6700 FT-IR spectrometer (Thermo Fisher Scientific).

### 4.3. Synthesis of PDI-CH_3_/CuO Nanocomposites

PDI-CH_3_/CuO nanocomposite was prepared according to a simple one-step precipitation route. An amount of 3 mL of 5 mM copper acetate (Cu(CH_3_COO)_2_), 10 μL glacial acetic acid and 5 mL 1 mg/mL PDI-CH_3_ were added in a round-bottomed flask. Then, the mixture was vigorously stirred and heated to 100 °C for approximately 15 min. Subsequently, 1 mL 0.5 M NaOH was quickly added into the above boiling solution and kept at 100 °C for 5 min. Finally, the precipitate was centrifuged, washed with distilled water and ethanol several times and redispersed into 3 mL of ultrapure water. The PDI-CH_3_/CuO nanocomposite dispersion solution was blackish-red emulsion. As control experiment, CuO nanomaterials were also prepared through above method. The CuO nanomaterials dispersion solution took on black-red color.

### 4.4. Preparation of Modified Electrodes

Initially, the GCE (diameter 3 mm) was polished with 0.3 μm γ-alumina powder and then ultrasonically cleaned with water and ethanol in sequence. Before modification, the prepared PDI-CH_3_/CuO was sonicated for 3 min. Then, 8 μL of that suspension was dropped onto the GCE surface and dried at room temperature. The PDI-CH_3_/CuO-modified GCE was further immersed in DA solution with different concentrations for 20 min at room temperature and rinsed with ultrapure water. Finally, the Model RFL-2 ECL analyzer (Xi’an Remex Instrument Co., Ltd., China) with electrochemical workstation CHI 660B (Shanghai CHI Instruments Co., China) was used to record the ECL signals in 5 mL 0.1 M PBS buffer (pH 6.5) containing 0.15 M K_2_S_2_O_8_, and the cycle scanning potential was from 0 to −1.0 V at a scan rate of 0.1 V/s. The photomultiplier tube voltage was set at 650 V. PDI-CH_3_ self-assembly material emitted strong ECL at −0.33 V in presence of coreactant K_2_S_2_O_8_. The ECL was quenched by CuO nanomaterials through ECL-RET mechanism. In addition, CuO nanomaterials covered on the PDI-CH_3_ self-assembly material also impeded the effective collision between PDI-CH_3_ and K_2_S_2_O_8_ and decreased the ECL intensity. After the PDI-CH_3_/CuO-modified GCE was reacted with DA, CuO nanomaterials were reduced into Cu^+^ due to the reductive property of DA. Then, CuO nanomaterials were reduced, ECL-RET decreased and the effective collision between PDI-CH_3_ and K_2_S_2_O_8_ increased, which led to the recovery of ECL. The difference in ECL intensity was taken as detection signal for DA (Scheme 1).

**Figure 8:**
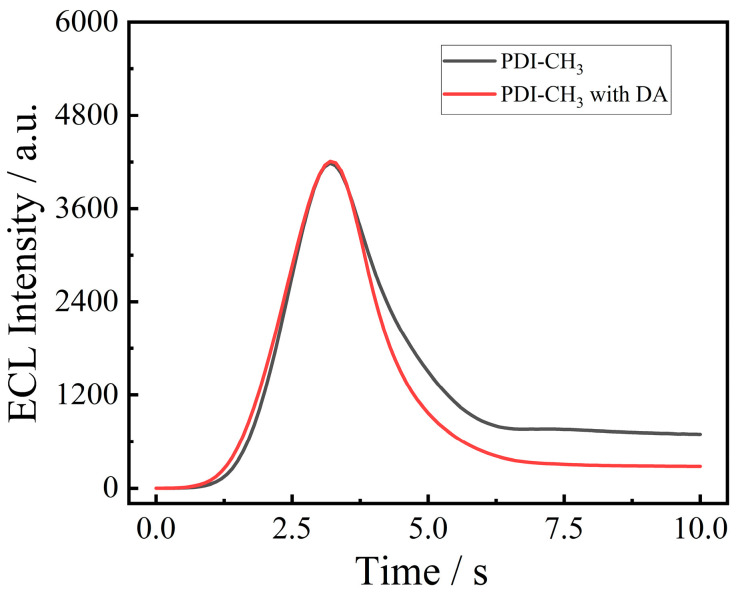
The ECL curves of PDI-CH_3_-modified GCE before (black line) and after (red line) treatment with DA recorded in 0.1 M PBS buffer (pH 6.5) containing 0.15 M K_2_S_2_O_8_. The cycle scanning potential was from 0 to −1.0 V at a scan rate of 0.1 V/s.

As control experiment, the ECL experiments of PDI-CH_3_-modified GCE before and after treatment with DA were performed (Figure 8). After treatment with DA (red line), there was no obvious ECL change for PDI-CH_3_-modified GCE. The results demonstrated that the CuO nanomaterial was indispensable in this work.

## 5. Conclusions

In this work, PDI-CH_3_ self-assembly nanomaterials were integrated together with CuO nanomaterials as an ECL platform for DA detection. PDI-CH_3_ self-assembly material emitted strong ECL at −0.33 V with coreactant K_2_S_2_O_8_. The ECL could be quenched by CuO nanomaterials through the ECL-RET mechanism. In addition, CuO nanomaterials covered on the PDI-CH_3_ self-assembly material also impeded the effective collision between PDI-CH_3_ and K_2_S_2_O_8_ and decreased the ECL intensity. In the presence of DA, CuO nanomaterials were reduced into Cu^+^ due to the reductive property of DA. Then, CuO nanomaterials were reduced, ECL-RET decreased and the effective collision between PDI-CH_3_ and K_2_S_2_O_8_ increased, which led to the recovery of ECL. With the increase of *C*_DA_, the ECL intensity increased drop-wisely. The difference in the ECL intensity was taken as a detection signal for DA. This ECL sensor exhibited a sensitive linear response to DA from 10^−12^ M to 10^−8^ M with a low detection limit of 0.20 pM. Compared with other DA sensors, the constructed ECL sensor exhibited higher sensitivity. In addition, the constructed ECL sensor exhibited good practicability in a human serum sample.

## Figures and Tables

**Table 1:** The comparison of different methods for DA detection.

Method	Linear Range (M)	LOD	Refs.
HPLC-FL	10^−6^–10^−5^	0.22 µM	[51]
Fluorescence	10^−6^–3 × 10^−5^	0.1 µM	[52]
Electrochemistry	10^−11^–10^−4^	4.1 pM	[50]
Electrochemistry	3 × 10^−8^–8 × 10^−5^	0.012 µM	[54]
ECL	6 × 10^−8^–1.6 × 10^−6^	0.028 µM	[53]
ECL	1 × 10^−8^–10^−3^	0.0082 µM	[22]
ECL	1 × 10^−13^–5.0 × 10^−9^	0.033 pM	[55]
ECL	10^−12^–10^−8^	0.20 pM	This work

**Table 2:** Determination of DA in human serum sample.

Samples	Added *C*_DA_ (pM) After Dilution	*C*_DA_ Found (pM) After Dilution	RSD (%)	Recovery Rate (%)
Human serum sample	0	0.316	0.27%	/
	10	9.55	4.7%	92.6%
	100	106.1	3.5%	105.8%
	1000	933	2.8%	93.3%

## Data Availability

The data that support the findings of this study are available from the corresponding author upon reasonable request.

## References

[1] Dong Z., Xia S., Alboull A.M.A., Mostafa I.M., Abdussalam A., Zhang W., Han S., Xu G. (2024). Bimetallic CoMoO_4_ Nanozymes Enhanced Luminol Chemiluminescence for the Detection of Dopamine. ACS Appl. Nano Mater..

[2] Zhang W., Zhang H., Li C., Shang L., Ma R., Jia L., Li X., Li B., Wang H. (2024). Dual-mode electrochemical and electrochemiluminescence detection of dopamine based on perylene diimide self-assembly material. Microchim. Acta.

[3] Li H., Zhang Y., Deng Z., Lu B., Ma L., Wang R., Wang X., Jiao Z., Wang Y., Zhou K. (2024). Constructing a Hydrophilic Microsensor for High-Antifouling Neurotransmitter Dopamine Sensing. ACS Sens..

[4] Lan Y., Yuan F., Fereja T.H., Wang C., Lou B., Li J., Xu G. (2019). Chemiluminescence of Lucigenin/Riboflavin and Its Application for Selective and Sensitive Dopamine Detection. Anal. Chem..

[5] Kamal Eddin F.B., Fen Y.W. (2020). The Principle of Nanomaterials Based Surface Plasmon Resonance Biosensors and Its Potential for Dopamine Detection. Molecules.

[6] Thakuri A., Banerjee M., Chatterjee A. (2024). Polydiacetylene Liposome-Based Dual-Output Optical Sensor for ppb Level Detection of Dopamine in Solution and Solid Phases. Langmuir.

[7] Feng J.-J., Guo H., Li Y.-F., Wang Y.-H., Chen W.-Y., Wang A.-J. (2013). Single Molecular Functionalized Gold Nanoparticles for Hydrogen-Bonding Recognition and Colorimetric Detection of Dopamine with High Sensitivity and Selectivity. ACS Appl. Mater. Interfaces.

[8] Yue J.-Y., Song L.-P., Wang Y.-T., Yang P., Ma Y., Tang B. (2022). Fluorescence/Colorimetry/Smartphone Triple-Mode Sensing of Dopamine by a COF-Based Peroxidase-Mimic Platform. Anal. Chem..

[9] Swain S., Jena A.K., Mohanta T. (2023). Ratiometric Fluorescence/Colorimetry/Smartphone Triple-Mode Real-Time Detection of Doxorubicin by Nitrogen-Doped Carbon Dots. ACS Appl. Opt. Mater..

[10] Shen H., Jang B., Park J., Mun H.-j., Cho H.-B., Choa Y.-H. (2022). In Situ Synthesis of a Bi_2_Te_3_-Nanosheet/Reduced-Graphene-Oxide Nanocomposite for Non-Enzymatic Electrochemical Dopamine Sensing. Nanomaterials.

[11] Li R., Guo W., Zhu Z., Zhai Y., Wang G., Liu Z., Jiao L., Zhu C., Lu X. (2023). Single-Atom Indium Boosts Electrochemical Dopamine Sensing. Anal. Chem..

[12] Gong S., Qin A., Zhang Y., Tian J., Li M., Liang Y., Xu X., Wang Z., Wang S. (2023). Construction of a Flavonol-Based Fluorescent Probe with a Large Stokes Shift for Highly Selective and Rapid Monitoring of H_2_S in Water, Foodstuff, and Living Systems. ACS Sustain. Chem. Eng..

[13] Du W., Shen Z., Liang Y., Gong S., Meng Z., Li M., Wang Z., Wang S. (2023). A Highly Effective “Naked Eye” Colorimetric and Fluorimetric Curcumin-Based Fluorescent Sensor for Specific and Sensitive Detection of H_2_O_2_ in Vivo and in Vitro. Analyst.

[14] Qing X., Wang Y., Zhang Y., Ding X., Zhong W., Wang D., Wang W., Liu Q., Liu K., Li M. (2019). Wearable Fiber-Based Organic Electrochemical Transistors as a Platform for Highly Sensitive Dopamine Monitoring. ACS Appl. Mater. Interfaces.

[15] Zhao M., Chen A.-Y., Huang D., Zhuo Y., Chai Y.-Q., Yuan R. (2016). Cu Nanoclusters: Novel Electrochemiluminescence Emitters for Bioanalysis. Anal. Chem..

[16] Bushira F.A., Kitte S.A., Xu C., Li H., Zheng L., Wang P., Jin Y. (2021). Two-Dimensional-Plasmon-Boosted Iron Single-Atom Electrochemiluminescence for the Ultrasensitive Detection of Dopamine, Hemin, and Mercury. Anal. Chem..

[17] Huo X.-L., Zhang N., Yang H., Xu J.-J., Chen H.-Y. (2018). Electrochemiluminescence Resonance Energy Transfer System for Dual-Wavelength Ratiometric miRNA Detection. Anal. Chem..

[18] Xia M., Zhou F., Feng X., Sun J., Wang L., Li N., Wang X., Wang G. (2021). A DNAzyme-Based Dual-Stimuli Responsive Electrochemiluminescence Resonance Energy Transfer Platform for Ultrasensitive Anatoxin-a Detection. Anal. Chem..

[19] Chen M.-M., Gao H., Ge Z.-B., Zhao F.-J., Xu J.-J., Wang P. (2024). Ultrasensitive Electrochemiluminescence Sensor Utilizing Aggregation-Induced Emission Active Probe for Accurate Arsenite Quantification in Rice Grains. J. Agric. Food Chem..

[20] Zhang S., Zheng H., Chen Y., Yi H., Dai H., Hong Z., Lin Y. (2019). Electrochemiluminescence Resonance Energy Transfer between Ru(bpy)_3_^2+^ and CdZnSe@ZnSe Quantum Dots for Ovarian Cancer Biomarker Detection. ACS Appl. Nano Mater..

[21] Wu M.-S., Shi H.-W., He L.-J., Xu J.-J., Chen H.-Y. (2012). Microchip Device with 64-Site Electrode Array for Multiplexed Immunoassay of Cell Surface Antigens Based on Electrochemiluminescence Resonance Energy Transfer. Anal. Chem..

[22] Li M., Wang C., Liu D. (2020). A novel “off-on” electrochemiluminescence sensor based on highly efficient resonance energy transfer in C-g-C_3_N_4_/CuO nanocomposite. Anal. Chim. Acta.

[23] Zhang B., Zhang F., Zhang P., Shen D., Gao X., Zou G. (2019). Ultrasensitive Electrochemiluminescent Sensor for MicroRNA with Multinary Zn–Ag–In–S/ZnS Nanocrystals as Tags. Anal. Chem..

[24] Fu L., Zhang B., Long X., Fu K., Gao X., Zou G. (2019). Promising Electrochemiluminescence from CuInS2/ZnS Nanocrystals/Hydrazine via Internal Cu(I)/Cu(II) Couple CyclingClick to copy article link. Anal. Chem..

[25] Cheng L., Liu X., Lei J., Ju H. (2010). Low-Potential Electrochemiluminescent Sensing Based on Surface Unpassivation of CdTe Quantum Dots and Competition of Analyte Cation to Stabilizer. Anal. Chem..

[26] He S., Li W., Li M., Liu Z., Sun X., Ding Z. (2024). Electrochemiluminescence of N,N′-Dimethylformamide Passivated Black Phosphorus Quantum Dots. Langmuir.

[27] Zhang W., Song Y., Wang Y., He S., Shang L., Ma R., Jia L., Wang H. (2020). A perylenetetracarboxylic dianhydride and aniline-assembled supramolecular nanomaterial with multi-color electrochemiluminescence for a highly sensitive label-free immunoassay. J. Mater. Chem. B.

[28] Zou G., Ju H. (2004). Electrogenerated chemiluminescence from a CdSe nanocrystal film and its sensing application in aqueous solution. Anal. Chem..

[29] Kovalchuk E.P., Reshetnyak O.V., Chernyak A.O., Kovalyshyn Y.S. (1999). Electrochemiluminescence on np1-metals: 1. The analysis of chemiluminescent reactions. Electrochim. Acta.

[30] Zhang W., Song Y., He S., Shang L., Ma R., Jia L., Wang H. (2019). Perylene diimide as a cathodic electrochemiluminescence luminophore for immunoassays at low potentials. Nanoscale.

[31] Zhang W., Song Y., Wang Y., Gong Y., Shang L., Ma R., Jia L., Xue Q., Du Y., He S. (2020). Perylene Dianhydride and Perylene Diimide Luminophores Integrated with Gold Nanoparticles for Dual-Potential Electrochemiluminescence Ratiometric Immunosensors. ACS Appl. Nano Mater..

[32] Song Y., Zhang W., He S., Shang L., Ma R., Jia L., Wang H. (2019). Perylene Diimide and Luminol as Potential-Resolved Electrochemiluminescence Nanoprobes for Dual Targets Immunoassay at Low Potential. ACS Appl. Mater. Interfaces.

[33] Zhang W., Cui X., Li F., Yan W., Wang Y., Shang L., Ma R., Jia L., Li C., Wang H. (2022). Perylene diimide and g-C_3_N_4_ nanosheet as potential-resolved cathode luminophores for ultrasensitive ratiometric electrochemiluminescence immunosensor. Sens. Actuators B-Chem..

[34] Wang Y., Li Y., Zhang W., Yin P., Shang L., Ma R., Jia L., Xue Q., He S., Wang H. (2021). Lowly-aggregated perylene diimide as a near-infrared electrochemiluminescence luminophore for ultrasensitive immunosensors at low potentials. Analyst.

[35] Lei Y.M., Huang W.X., Zhao M., Chai Y.Q., Yuan R., Zhuo Y. (2015). Electrochemiluminescence resonance energy transfer system: Mechanism and application in ratiometric aptasensor for lead ion. Anal. Chem..

[36] Zhong H., Yuan Y., Qing M., Zuo J., Li Y., Bai L. (2023). Electrochemiluminescence Aptasensor for HER_2_-ECD Detection Based on Resonance Energy Transfer between AuPt@ZIF-67 and AuNPs/g-C_3_N_4_/PDDA Composites. ACS Appl. Nano Mater..

[37] Feng Q., Qin L., Dou B., Han X., Wang P. (2022). Plasmon-Tunable Ag@Au Bimetallic Core–Shell Nanostructures to Enhance the Electrochemiluminescence of Quantum Dots for MicroRNA Sensing. ACS Appl. Nano Mater..

[38] Jiang D., Du X., Liu Q., Zhou L., Qian J., Wang K. (2015). One-Step Thermal-Treatment Route to Fabricate Well-Dispersed ZnO Nanocrystals on Nitrogen-Doped Graphene for Enhanced Electrochemiluminescence and Ultrasensitive Detection of Pentachlorophenol. ACS Appl. Mater. Interfaces.

[39] Li J., Lai W., Ma C., Zhao C., Li P., Jiang M., Wang M., Chen S., Hong C. (2022). MnO_2_ Nanosheet/Polydopamine Double-Quenching Ru(bpy)_3_^2+^@TMU-3 Electrochemiluminescence for Ultrasensitive Immunosensing of Alpha-Fetoprotein. ACS Appl. Nano Mater..

[40] Song X., Zhao L., Ren X., Feng T., Ma H., Wu D., Li Y., Luo C., Wei Q. (2022). Highly Efficient PTCA/Co_3_O_4_/CuO/S_2_O_8_^2–^ Ternary Electrochemiluminescence System Combined with a Portable Chip for Bioanalysis. ACS Sens..

[41] Song C., Li X., Hu L., Shi T., Wu D., Ma H., Zhang Y., Fan D., Wei Q., Ju H. (2020). Quench-Type Electrochemiluminescence Immunosensor Based on Resonance Energy Transfer from Carbon Nanotubes and Au-Nanoparticles-Enhanced g-C_3_N_4_ to CuO@Polydopamine for Procalcitonin Detection. ACS Appl. Mater. Interfaces.

[42] Williams M.E., Murray R.W. (1998). Perylene Polyether Hybrids: Highly Soluble, Luminescent, Redox-Active Dyes. Chem. Mater..

[43] Backes C., Schmidt C.D., Rosenlehner K., Hauke F., Coleman J.N., Hirsch A. (2010). Nanotube Surfactant Design: The Versatility of Water-Soluble Perylene Bisimides. Adv. Mater..

[44] Kou T., Si C., Gao Y., Frenzel J., Wang H., Yan X., Bai Q., Eggeler G., Zhang Z. (2014). Large-scale synthesis and catalytic activity of nanoporous Cu-O system towards CO oxidation. RSC Adv..

[45] Pendashteh A., Mousavi M.F., Rahmanifar M.S. (2013). Fabrication of anchored copper oxide nanoparticles on graphene oxide nanosheets via an electrostatic coprecipitation and its application as supercapacitor. Electrochim. Acta.

[46] Wang P., Ng Y.H., Amal R. (2013). Embedment of anodized p-type Cu_2_O thin films with CuO nanowires for improvement in photoelectrochemical stability. Nanoscale.

[47] Sun X., Li C., Wu Z., Han L., Zhang W., Han D., Niu L. (2024). Perylene Derivative Self-Assembly Material for Photoelectrochemical Biosensing. ACS Appl. Nano Mater..

[48] Zhang W., Sun X., Liu H., Shang L., Ma R., Li X., Jia L., He S., Li C., Wang H. (2024). Self-Powered Photoelectrochemistry Biosensor for Ascorbic Acid Determination in Beverage Samples Based on Perylene Material. Molecules.

[49] Zhang H., Li B., Wang R., Miao Q., Cui X., Shang L., Ma R., Jia L., Li C., Li F. (2023). Perylene derivative and persulfate as highly efficient electrochemical system for constructing sensitive amperometric aptasensor. Talanta.

[50] Cui X., Geng H., Zhang H., Sun X., Shang L., Ma R., Jia L., Li C., Zhang W., Wang H. (2024). A perylene diimide electrochemical probe with persulfate as a signal enhancer for dopamine sensing. Analyst.

[51] Chaiendoo K., Ittisanronnachai S., Promarak V., Ngeontae W. (2019). Polydopamine-coated carbon nanodots are a highly selective turn-on fluorescent probe for dopamine. Carbon.

[52] Tan X., Zhang P., Ye C., Min Y., Li Q., Wang Y. (2020). Signal-on photoluminescent detection of dopamine with carbon dots-MnO_2_ nanosheets platform based on inner filter effect. Dyes Pigments.

[53] Pan Q., Xu Z., Deng S., Zhang F., Li H., Cheng Y., Wei L., Wang J., Zhou B. (2019). A mechanochemically synthesized porous organic polymer derived CQD/chitosan–graphene composite film electrode for electrochemiluminescence determination of dopamine. RSC Adv..

[54] Deng K., Li X., Huang H. (2016). Synthesis of a novel triad hybrid of noncovalent-assembled nickel (II) norcorrole on graphene oxide encapsulated multiwalled carbon nanotubes and its application. Electrochim. Acta.

[55] Zuo F., Jin L., Fu X., Zhang H., Yuan R., Chen S. (2017). An electrochemiluminescent sensor for dopamine detection based on a dual-molecule recognition strategy and polyaniline quenching. Sens. Actuators B-Chem..

